# Clusterin protects against HFpEF by inhibiting UCHL1-mediated NLRP3 deubiquitylation and inflammasome activation

**DOI:** 10.3389/fphar.2025.1704023

**Published:** 2026-01-13

**Authors:** Jiangling Yu, Xiaoxu Kang, Rui Chang, Cheng Zhang, Song Yang, Lang Chen, Xinbo Wang, Bing Hu, Zixuan Wang, Lili Gong, Lihong Liu

**Affiliations:** 1 Institute of Clinical Medical Sciences, China-Japan Friendship Hospital, Capital Medical University, Beijing, China; 2 Institute of Clinical Medical Sciences, China-Japan Friendship Hospital, Beijing, China; 3 Department of Pharmacy, China-Japan Friendship Hospital, Beijing, China; 4 China-Japan Friendship Hospital (Institute of Clinical Medical Sciences), Chinese Academy of Medical Sciences & Peking Union Medical College, Beijing, China

**Keywords:** clusterin, HFpEF, NLRP3, ubiquitination, UCHL1

## Abstract

**Background:**

Heart failure with preserved ejection fraction (HFpEF) poses a serious threat to human health, but effective treatment strategies remain limited. Clusterin (CLU) is a multifunctional glycoprotein implicated in inflammation and tissue remodeling, but its role in HFpEF pathogenesis is not fully understood.

**Methods:**

The effects of CLU in a murine HFpEF model were investigated by adeno-associated virus (AAV)-mediated overexpression and liver-specific knockout approaches. Cardiac function in mice was evaluated by echocardiography, and myocardial inflammation and fibrosis were assessed using Masson’s trichrome staining, real-time qPCR, and Western blot analysis. Protein interactions were identified by immunoprecipitation–mass spectrometry (IP-MS).

**Results:**

AAV-mediated CLU overexpression significantly improved diastolic function and reduced myocardial inflammation and fibrosis in HFpEF mice, whereas liver-specific CLU knockout aggravated disease progression. *In vitro*, CLU overexpression attenuated inflammatory responses and collagen production in injured cardiomyocytes. Mechanistically, CLU was found to interact with the deubiquitinase UCHL1. CLU overexpression reduced UCHL1 expression, thereby enhancing ubiquitination and degradation of NLRP3, leading to suppression of inflammasome activation and inflammation. Furthermore, treatment with a synthetic CLU-derived peptide markedly alleviated cardiac fibrosis and inflammation in HFpEF mice.

**Conclusion:**

This study reveals a novel regulatory mechanism through which CLU alleviates HFpEF by modulating the UCHL1–NLRP3 signaling axis. The findings provide new insight into the anti-inflammatory and anti-fibrotic roles of CLU and suggest that CLU and its derived peptide hold translational potential as therapeutic candidates for HFpEF.

## Graphical Abstract

**Figure:**
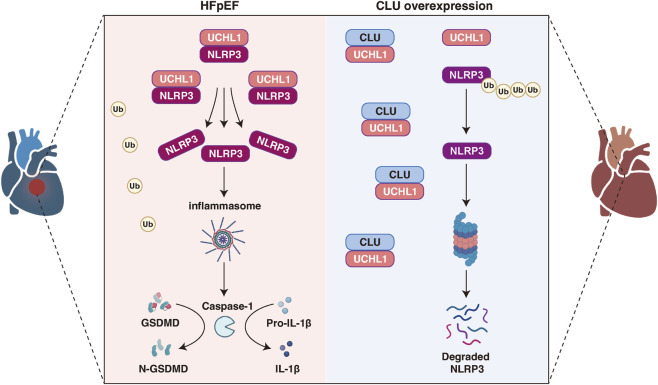

## Highlights

- Clusterin (CLU) exerted cardioprotective effects in HFpEF by modulating cardiac inflammation and fibrosis.
- CLU interacted with the deubiquitinating enzyme UCHL1 and promoted NLRP3 degradation via enhanced ubiquitination, thereby inhibiting inflammasome activation.
- Hepatocyte-specific knockdown of CLU aggravated HFpEF progression, highlighting the importance of liver–heart crosstalk in disease development.
- A D-amino acid-modified CLU-derived peptide (CLU-113D) showed therapeutic efficacy in a mouse model of HFpEF.
- This study identified a novel CLU–UCHL1–NLRP3 regulatory axis, providing a potential target for peptide-based therapies in HFpEF.

## 1 Introduction

Heart failure (HF), also known as congestive heart failure, is a condition in which the heart is unable to pump sufficient blood to meet the body’s needs. It is the leading cause of death from cardiovascular diseases. Heart failure includes heart failure with preserved ejection fraction (HFpEF) and heart failure with reduced ejection fraction (HFrEF). HFpEF accounts for 50% of all heart failure cases, and 70% of heart failure patients over 65 are HFpEF (Dunlay et al., 2017; Tang et al., 2021; Ziaeian and Fonarow, 2016). It primarily affects olders and is often accompanied by other health issues such as diabetes, hypertension, and obesity. At present, the challenge in treating HFpEF lies in developing effective strategies, as the symptoms and quality of life of patients with HFpEF are comparable to those with reduced ejection fraction, but specific drugs for HFrEF are ineffective in this case, the discovery of therapeutic targets for HFpEF serves as a crucial strategy to improve patients’ quality of life and reduce mortality (Vaishnav and Sharma, 2022).

The pathogenesis of HFpEF is multifactorial and incompletely understood. Emerging evidence suggests that systemic inflammation, oxidative stress, and endothelial dysfunction contribute to myocardial remodeling and fibrosis, which are key features of HFpEF. Microvascular rarefaction, immune activation, and metabolic dysregulation further exacerbate disease progression (Boulet et al., 2024; Capone et al., 2022; Schiattarella and Hill, 2021; Wu et al., 2022). Current treatment strategies include monitoring patients’ weight, limiting sodium and calorie intake, and strengthening exercise to lose weight. Drugs such as SGLT2 inhibitors, used in patients with type 1 diabetes, a history of diabetic ketoacidosis, or an estimated glomerular filtration rate (EGFR) of <20 mL/min/1.73 m^2^, are also employed (Nassif et al., 2021; Requena-Ibanez et al., 2023). However, there are still no specific effect drugs for the treatment of HFpEF.

Clusterin (CLU), also known as Apolipoprotein J (ApoJ), is a kind of secreted mammalian chaperone protein. In addition to functioning as an apolipoprotein, it also acts as an extracellular chaperone to promote cell survival, sperm maturation, complement inhibition, and cell differentiation (Rodríguez-Rivera et al., 2021; Satapathy and Wilson, 2021). The expression of CLU has been reported to show upregulated in various metabolic diseases, such as hypertension, diabetes, diabetic nephropathy, and other metabolic diseases (López Malizia et al., 2024; Praharaj et al., 2023; Wilson et al., 2022). Additionally, it has been observed to dynamically correlate with acute myocardial infarction. CLU is also involved in cholesterol efflux and reverse transport, and is an important component of high-density lipoprotein (Wittwer and Bradley, 2021). Numerous studies have demonstrated the presence of inflammation, mitochondrial damage, and abnormal lipid metabolism in patients with HFpEF (Deng et al., 2021; Schauer et al., 2024; Schiattarella et al., 2021). Therefore, we speculate that CLU may potentially play an important role in the disease progression of HFpEF.

NLRP3 is an important component of inflammasomes, which contain the NOD-like receptor NLRP3, ASCs, and the effector protein caspase-1, playing an important role in macrophage innate immunity (Li S. et al., 2024; Song et al., 2020). NLRP3 initiates transcription and inflammasome assembly of downstream signaling proteins, which are transformed from inactive homomers to active multimeric inflammasomes under the action of post-translational modifications (PTMs). Activation of NLRP3 inflammasomes induces self-cleavage and activation of caspase-1 and promotes the maturation and secretion of IL-1β and IL-18 in macrophages (Elliott and Sutterwala, 2015; Xia et al., 2023). Higher levels of IL-1β and IL-18 have been reported in blood samples from patients with HFpEF compared with age-matched non-HF patients, suggesting activation of the NLRP3-mediated inflammatory cascade during the pathogenesis of HFpEF (Toldo and Abbate, 2024). However, the relationship between CLU and NLRP3 remains unclear.

Herein, we aimed to explore the potential role of CLU in the development of HFpEF and investigate the underlying molecular mechanisms, with particular focus on its interaction with the deubiquitinating enzyme UCHL1 and its impact on NLRP3 inflammasome activity.

## 2 Materials and methods

### 2.1 Experimental animals and HFpEF model

Six-week-old male C57BL/6J mice were obtained from Vital River Laboratory Animal Technology (Beijing, China). All mice were housed under specific pathogen-free (SPF) conditions with controlled temperature (22 °C–24 °C), humidity (50%–60%), and a 12 h light/dark cycle. Standard rodent chow and water were provided *ad libitum*. All experimental procedures were approved by the China–Japan Friendship Hospital Institutional Animal Ethics Committee (Beijing, China).

To establish the HFpEF model, mice were randomly assigned to either a standard chow diet or a high-fat diet (HFD, 60% kcal, Research Diet, Cat# D12492) combined with Nω-nitro-L-arginine methyl ester (_L_-NAME) (0.5 g/L in drinking water) for 5 weeks.

### 2.2 Echocardiography in mice

Cardiac function was assessed using a high-resolution small animal echocardiography system (FujiFilm VisualSonics, Vevo F2). Mice were anesthetized with 1%–2% isoflurane in oxygen and placed on a heated platform to maintain body temperature at 36 °C–37 °C. Heart rate was carefully monitored and maintained at 400–500 bpm to ensure accurate measurement of systolic and diastolic function. Two-dimensional and M-mode images were acquired to evaluate left ventricular ejection fraction (LVEF), fractional shortening (LVFS), and diastolic function parameters including E/A and E/E′ ratios.

### 2.3 Evaluation of the effects of CLU overexpression on HFpEF in mice

To induce CLU overexpression *in vivo*, adeno-associated virus serotype 9 (AAV9) vectors carrying the mouse CLU gene (AAV9-CLU) or green fluorescent protein (AAV9-GFP) as a control were obtained from HANBio Biotechnology (ShangHai, China). Mice were randomly assigned to the CLU or control groups and administered a single intravenous dose of AAV9 (1 × 10^10^ genome copies per mouse) via the tail vein. CLU expression was verified by assessing mRNA levels (qPCR), protein expression (Elisa). All experiments were conducted in accordance with institutional animal care guidelines and biosafety regulations.

The experimental groups were as follows: Control, HFpEF, HFpEF + CLU AAV, and HFpEF + GFP AAV (n = 6 per group). Mice in the Control group were fed a standard chow diet, and HFpEF was induced in the other groups by HFD feeding combined with _L_-NAME administration. Cardiac function was evaluated by echocardiography at week 5.

### 2.4 Evaluation of the effects of liver-specific CLU knockout on HFpEF in mice

To generate liver-specific CLU knockout mice, CLU^Flox/Flox^ (CLU^fl/fl^) conditional knockout mice and liver-specific Cre-expressing mice were purchased from Cyagen Biosciences (China). The Cre-expressing mice utilized the Alb-Cre system, in which Cre recombinase is driven by the albumin promoter, allowing liver-specific expression in hepatocytes.

CLU^fl/fl^ mice were first crossed with Alb-Cre mice to obtain heterozygous offspring with the genotype CLU^Flox/WT^; Cre^+^. These heterozygotes were then intercrossed, and the progeny were genotyped using RT-qPCR to identify CLU^Flox/Flox^; Cre^+^ homozygous mice, which were designated as liver-specific CLU knockout (CLU^lko^) mice.

The experimental groups were as follows: Control, CLU^fl/fl^, CLU^lko^ (n = 6 per group). Mice in the Control group were fed a standard chow diet, and HFpEF was induced in the other groups by HFD feeding combined with _L_-NAME administration. Cardiac function was evaluated by echocardiography at week 5.

### 2.5 Evaluation of the therapeutic effects of CLU peptide on HFpEF

Mice were divided into three groups: Control, HFpEF, and HFpEF-CLU peptide. Mice in the control group received standard chow and water, while those in the HFpEF and HFpEF-CLU peptide groups were administered _L_-NAME in drinking water combined with a HFD. In addition, mice in the HFpEF-CLU peptide group received daily oral administration of CLU peptide at a dose of 5 mg/kg. After 5 weeks of treatment, cardiac function was evaluated by echocardiography. Following confirmation of successful HFpEF induction, mice were sacrificed for sample collection. Blood, heart, liver, lung, and tibia were harvested, and the weights of the heart and lung were recorded.

### 2.6 Cell culture

THP-1 human monocytic cells were cultured in RPMI-1640 medium supplemented with 10% fetal bovine serum, 100 U/mL penicillin, and 100 μg/mL streptomycin at 37 °C in a humidified atmosphere with 5% CO_2_. AC16 human cardiomyocytes were maintained in DMEM/F12 medium containing 10% FBS and antibiotics under the same conditions.

### 2.7 “3-hit” cell model in AC16 cells

THP1 cells were first stimulated with PMA (100 ng/mL) for 48 h, then treated with LPS (200 ng/mL) for 6 h and NLRP3 activator ATP (2 mM, for 30 min), the medium was collected as macrophage conditioned medium (MCM). To neutralize IL-10, MCM was incubated with anti-IL-10 antibody for 12 h. AC16 cells were treated with isoproterenol (ISO, 20 μM) for 48 h and MCM with β-OHB (0.5 mM or 5 mM) for 24 h to mimic the 3-Hit-induced pathological cardiomyocyte hypertrophy and inflammation environment.

### 2.8 “2-hit” cell model in AC16 cells

The well-grown AC16 cardiomyocytes were spread in a six-well plate, and 200 ng/mL Ang II was added to the experimental group. After 12 h of treatment, the cells were collected and RNA was extracted. After reverse transcription, the expressions of inflammatory factors and fibrosis-related genes were detected by qPCR technology.

### 2.9 Immunoprecipitation–mass spectrometry (IP-MS)

AC16 cells were washed with cold PBS and lysed in NP40 buffer containing protease and phosphatase inhibitors (1 mL/1 × 10^7^ cells). Lysates were incubated on ice for 30 min and centrifuged at 12,000 g for 30 min at 4 °C. The supernatants were collected and divided into experimental (IP) and control (IgG) groups, with aliquots reserved as input controls. For Co-IP, 20 μL of Protein A/G Plus agarose beads were incubated with 2 μg anti-clusterin antibody (IP) or 0.1–0.2 μg non-immune IgG (control) overnight at 4 °C. Beads were washed 3–5 times with cold wash buffer and eluted with 2× SDS loading buffer at 99 °C for 10 min. The eluates were analyzed by SDS-PAGE and LC-MS/MS, and peptide identification was performed using data-dependent acquisition (DDA).

### 2.10 Adenoviral transfection

AC16 cells were transduced with adenoviruses carrying CLU (Ad-CLU) at the indicated multiplicity of infection (MOI) according to the manufacturer’s instructions (HANBio Biotechnology). Following transduction, cells were maintained under either normal culture conditions or subjected to the “double-damage” protocol for 48 h before subsequent analyses.

### 2.11 Histological analysis

Mouse heart tissues were collected and fixed in 10% neutral buffered formaldehyde for 24–48 h, followed by paraffin embedding. Tissue sections were prepared using a microtome.

Masson trichrome staining: Dewaxed rehydrated sections were stained serially to visualize collagen fibers: nuclei were stained with Weigert’s iron hematoxylin, cytoplasm and myofibrils were stained with acid violet-carmine, and collagen fibers were stained with aniline blue. This was followed by differentiation processing, dehydration, hyalinization and sealing of the slices for final visualization under the microscope. Collagen deposition was assessed as blue-stained areas in the tissue.

Hematoxylin-eosin (H&E) staining: Sections were deparaffinized by xylene, rehydrated by a gradient ethanol series, stained first with hematoxylin to reveal the nuclei, and then with eosin to enhance the contrast between the cytoplasm and extracellular matrix. The stained sections were dehydrated and transparent, then coverslipped and observed under light microscope.

All histologic analyses were performed in a blinded manner and images were acquired by a light microscope equipped with a digital camera.

### 2.12 Western blot analysis

Heart tissues were sonicated in a lysis buffer containing 20 mM Tris (pH 7.4), 150 mM NaCl, 1 mM EDTA, 1 mM EGTA, 1% Triton, 0.1% sodium dodecyl sulfate, and a protease inhibitor cocktail. Protein samples were incubated with anti-CLU (Cell Signaling Technology, Cat# 34642), anti-NLRP3 (Proteintech, Cat# 68102-1-Ig), anti-Col1a2 (Abcam, Cat# ab308455), anti-UCHL1 (Proteintech, Cat# 14730-1-AP), anti-p62 (Proteintech, Cat# 18420-1-AP), anti-LC3B (Proteintech, Cat# 14600-1-AP). Horseradish peroxidase-coupled secondary antibodies were employed. After immunoblotting, films were scanned and detected using a Bio-Rad calibrated densitometer and band intensity was normalized to loading control α-Tublin (Proteintech, Cat# 14555-1-AP) or β-Actin (Cell Signaling Technology, Cat# 4967).

### 2.13 RT-qPCR

Total RNA was extracted from AC16 cells, THP-1 cells or heart tissue using TRIzol reagent (Takara, Japan). Complementary DNA (cDNA) was synthesized with Hiscript II reverse transcriptase (Vazyme, Cat# R323). Gene expression levels were quantified by real-time PCR using the QuantStudio 5 system (Thermo Fisher) and SYBR Green PCR mix (Vazyme, Cat# Q712). 18S or β-actin served as internal controls. The sequences of all primers used in the experiments are listed in Supplementary Table S1.

### 2.14 Statistical analysis

The distribution of the data was assessed for normality using the Shapiro–Wilk or Kolmogorov–Smirnov test. For data that did not conform to a normal distribution, non-parametric tests were applied, including the Mann–Whitney U test for comparisons between two groups and the Kruskal–Wallis test for multiple groups. For normally distributed data, comparisons between two groups were performed using Student’s t-test, while multiple-group comparisons were conducted using one-way analysis of variance (ANOVA) followed by the LSD *post hoc* test. Statistical analyses were conducted using GraphPad Prism 10 software, and data are presented as mean ± SD. Significance is expressed as *p < 0.05, **p < 0.01, ***p < 0.001, ****p < 0.0001, and ns p > 0.05.

## 3 Results

### 3.1 Overexpression of CLU attenuates the progression of HFpEF disease

**FIGURE 1:**
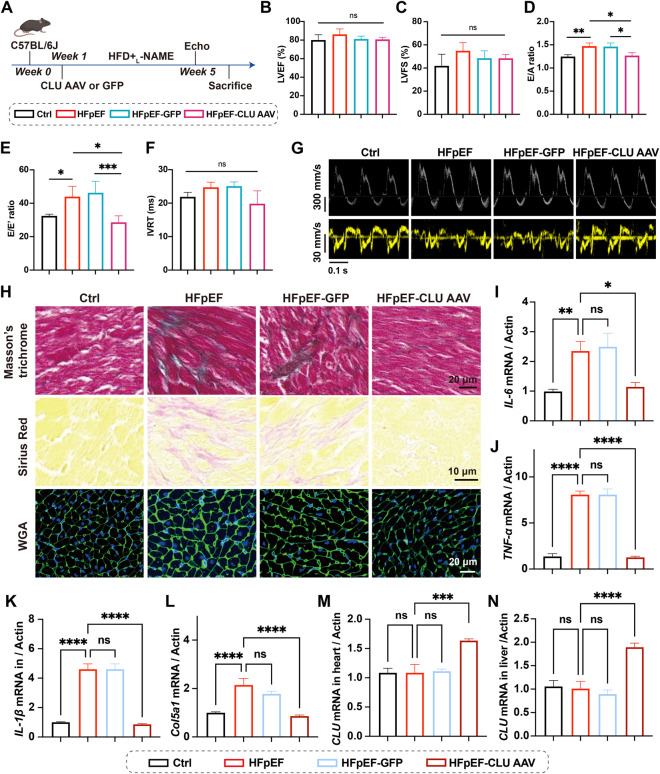
Overexpression of CLU attenuated the progression of HFpEF. **(A)** Schematic representation of the HFpEF mouse model induced by HFD + _L_-NAME, with intravenous injection of CLU adeno-associated virus (AAV) at week 1 to induce CLU overexpression. Percentages of **(B)** left ventricular ejection fraction (LVEF) and **(C)** left ventricular fractional shortening (LVFS), **(D)** E/A ratio **(E)** E/E′ ratio, and **(F)** isovolumic relaxation time of mice (n = 6). **(G)** Representative echocardiographic images of each group. **(H)** Representative Masson’s trichrome, Sirius red and WGA staining images of heart sections. mRNA expression levels of **(I)** IL-6, **(J)**TNF-α, **(K)** IL-1β, **(L)** Col5a1, and **(M)** CLU in heart tissues (n = 6). **(N)** CLU mRNA expression of Liver tissues (n = 6).

To understand the effect of CLU on HFpEF progression, AAV9 vectors that carrying CLU gene or green fluorescent protein (GFP) were constructed and were administered to the mice by intravenous injection (*I. V*) (Figure 1A). Mice were subjected to a “two-hit” HFpEF model induced by a combination of high-fat diet (HFD) and _L_-NAME-containing drinking water (Gao et al., 2024; Schiattarella et al., 2019). At week 5, body weight was recorded to evaluate systemic metabolic status. As expected, mice in the HFpEF, HFpEF-GFP, and HFpEF-CLU groups exhibited significantly higher body weight compared with the control group due to HFD + _L_-NAME exposure. However, no significant differences were observed among the three HFpEF groups, indicating that CLU overexpression did not influence body weight in this model (Supplementary Figure S1). Subsequently, the systolic and diastolic function of the heart were evaluated by echocardiography. During echocardiographic measurements, the heart rates of mice were maintained within the range of 400–500 bpm (Supplementary Figure S2A). The left ventricular ejection fraction (LVEF) of mice in all groups was higher than 50% and there was no significant difference (Figure 1B). The left ventricular fractional shortening (LVFS) of mice was also no significant change (ns, p > 0.05; Figure 1C). Both E/A ratio and E/E′ ratio were significantly increased in the HFpEF model group of mice compared to the control group (p = 0.0095, ** for E/A ratio; p = 0.03, * for E/E’ ratio; Figures 1D, E). In addition, no significant differences in isovolumic relaxation time (IVRT) were observed among groups (ns, p > 0.05; Figure 1F). Representative echocardiographic images are shown in Figure 1G. However, cardiac E/A and E/E′ were significantly decreased in the CLU overexpression group of mice, exhibiting comparable levels to those in the control group (p = 0.02 for E/A ratio; p = 0.02, * for E/E′ ratio), indicating that CLU overexpression could attenuate HFpEF progression. Furthermore, as shown in Figure 1H, Masson’s trichrome and Sirius red staining reveal markedly increased collagen deposition in the HFpEF group compared with the control group, whereas CLU overexpression (HFpEF-CLU AAV) substantially reduced myocardial fibrosis relative to the HFpEF and HFpEF-GFP groups (Supplementary Figure S3). In addition, WGA staining indicated pronounced cardiomyocyte hypertrophy in HFpEF mice, which was alleviated following CLU overexpression. Also, inflammatory and fibrotic factors (IL-6, TNF-α, IL-1β) and fibrotic factors (Col5a1) were significantly reduced in the cardiac tissues after overexpression of CLU, compared with HFpEF and HFpEF-GFP groups (Figures 1I–L). At last, overexpression of CLU in mice after injection of CLU AAV was confirmed, which were significantly increased in heart, liver and plasma in mice (Figures 1M,N; Supplementary Figure S4). Therefore, it could be concluded that overexpression of CLU attenuated cardiac diastolic impairment, cardiac inflammation and myocardial fibrosis caused by HFD + _L_-NAME diet.

### 3.2 Liver specific CLU knockout accelerates disease progression of HFpEF

**FIGURE 2:**
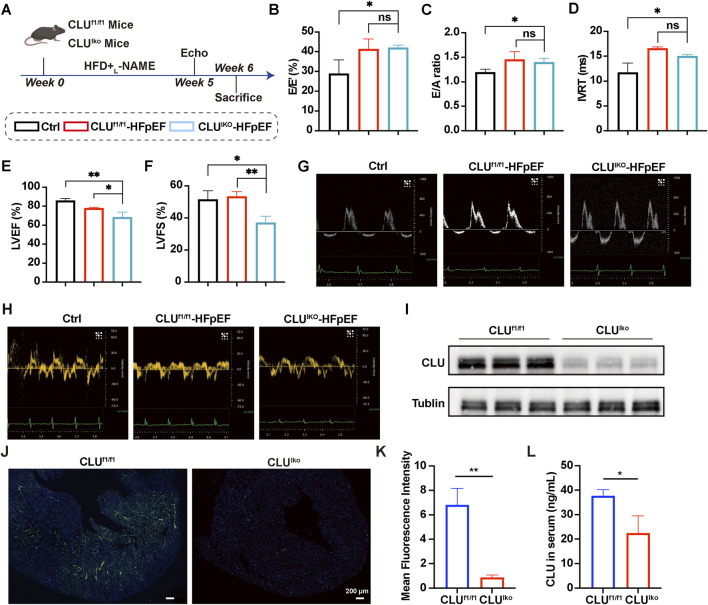
Liver CLU knockout aggravated HFpEF symptoms in mice. **(A)** Timeline of CLU^f1/f1^ and CLU^lko^ mice experiments. **(B)** E/A ratio, **(C)** E/E′ ratio, and **(D)** isovolumic relaxation time (IVRT) of mice. Percentage of **(E)** LVEF **(F)** LVFS (n = 6). **(G,H)** Representative echocardiographic images of each group. **(I)** Western blot detection of liver CLU expression. **(J)** Immunofluorescence staining of CLU expression in the hearts of CLU^f1/f1^ and CLU^lko^ mice. The green fluorescence comes from CLU protein. The blue fluorescence comes from nucleus. **(K)** Quantification of mean fluorescence intensity of CLU (green) in heart sections (n = 3) **(L)** The concentrations of CLU in serum of CLU^f1/f1^ and CLU^lko^ mice, respectively (n = 3).

The liver has been identified as the primary source of circulating CLU *in vivo* (Deng et al., 2021; Liang et al., 2024). To investigate the physiological role of hepatic CLU in the development of HFpEF, liver-specific CLU knockout mice (CLU^lko^) were generated by crossing CLU^fl/fl^ mice with Alb-Cre transgenic mice. Adult CLU^fl/fl^ and CLU^lko^ mice were then fed a high-fat diet (HFD) and administered _L_-NAME for 5 weeks to induce HFpEF phenotypes (Figure 2A). The heart rates of mice were recorded during echocardiographic measurements, which were maintained within the physiological range of 400–500 bpm (Supplementary Figure S2B). Compared with the normal control group, both CLU^fl/fl^ and CLU^lko^ mice displayed features of diastolic dysfunction, including elevated E/A ratio, E/E′ ratio, along with a modest decline in IVRT (Figures 2B–D). Notably, CLU^lko^ mice exhibited significantly lower LVEF and LVFS compared with CLU^fl/fl^ mice (Figures 2E,F), indicating more severe cardiac dysfunction and a progression toward heart failure. The representative echocardiographic images were shown in Figures 2G,H. Western blot analysis further confirmed a marked reduction of CLU protein expression in the liver tissues of CLU^lko^ mice compared with CLU^fl/fl^ controls (Figure 2I). Subsequently, we assessed CLU expression levels in both the heart tissue and serum of CLU^fl/fl^ and CLU^lko^ mice. As expected, CLU expression in the heart was significantly reduced in CLU^lko^ mice compared to CLU^fl/fl^ mice (Figure 2J). Quantitative analysis revealed that the mean fluorescence intensity in the hearts of CLU^lko^ mice was approximately 7-fold lower than that of CLU^fl/fl^ mice (Figure 2K). Moreover, CLU protein and mRNA levels in heart tissue were assessed by Western blotting and qPCR, respectively. Consistent with the immunofluorescence results, a marked decrease in CLU protein abundance was detected in CLU^lko^ hearts, and CLU mRNA expression was similarly reduced, further confirming the successful deletion of CLU in heart tissue (Supplementary Figure S5). In addition, the serum concentration of CLU was 37.7 ng/mL in CLU^fl/fl^ mice and 22.4 ng/mL in CLU^lko^ mice, indicating a significant reduction in circulating CLU levels in the CLU^lko^ group and confirming the liver as a major contributor to circulating and cardiac CLU (Figure 2L). Taken together, these results demonstrated that hepatic CLU deficiency leads to more severe cardiac dysfunction, thereby accelerating HFpEF progression and reinforcing the crucial role of CLU in cardioprotection.

### 3.3 CLU can relieve inflammation and fibrosis *in vitro*

The 3-HIT cell model is a widely accepted *in vitro* model for studying HFpEF. In this model, macrophages were first stimulated with LPS and ATP to generate macrophage-conditioned medium (MCM). Subsequently, AC16 cardiomyocytes were treated with both MCM and isoproterenol to mimic the inflammatory and neurohormonal stress characteristic of HFpEF (Deng et al., 2021). CLU adenovirus (ADV) was prepared and added to AC16 cells to overexpression of the CLU protein.

**FIGURE 3:**
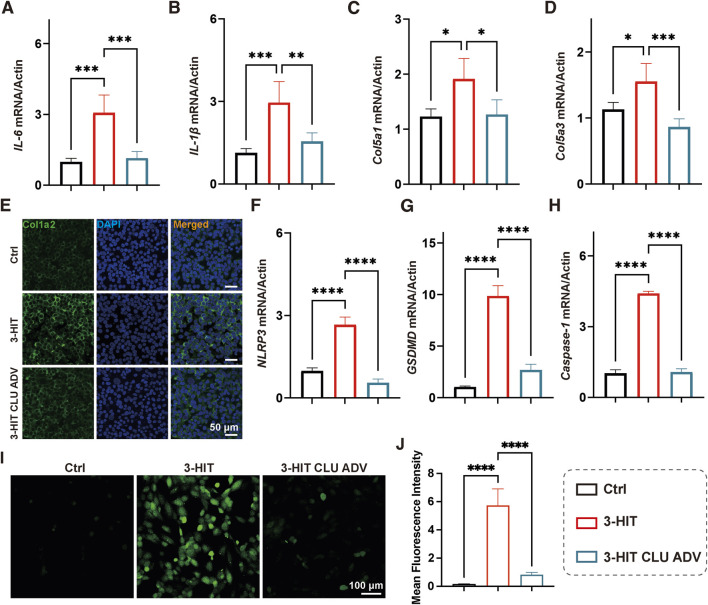
CLU alleviated inflammation and fibrosis in cardiomyocytes. The relative mRNA expression of **(A)** IL-6, **(B)** IL-1β, **(C)** Col5a1, and **(D)** Col5a3 in AC16 cells under 3-HIT stimulation with or without CLU overexpression (n = 3). **(E)** Representative confocal immunofluorescence images showing Col1a2 expression in AC16 cells. The relative mRNA expression of **(F)** NLRP3, **(G)** GSDMD, **(H)** Caspase-1 in AC16 cells treated with “3-HIT” and “3-HIT” plus CLU overexpression (n = 3). **(I)** Confocal fluorescence images of intracellular ROS detected using DCFH-DA. **(J)** Quantification of ROS fluorescence intensity (n = 3).

qPCR results showed that the “3-HIT” treatment significantly increased the expression of pro-inflammatory (IL-6, IL-1β) and fibrogenic factors (col5a1, col5a3), and CLU overexpression resulted in a significant reduction of these factors (Figures 3A–D). Consistently, immunofluorescence microscopy revealed increased Col1a2 expression under 3-HIT conditions, which was alleviated by CLU (Figure 3E). Given the importance of pyroptosis in HFpEF pathology, we further examined pyroptosis-related genes. The expression of NLRP3, GSDMD, and Caspase-1 was markedly elevated in AC16 cells following 3-HIT stimulation, indicating activation of pyroptotic cell death. CLU overexpression substantially suppressed the expression of these markers (Figures 3F–H), suggesting that CLU mitigates inflammation and fibrosis by reducing cellular pyroptosis. To complement these findings, intracellular oxidative stress was assessed using the DCFH-DA fluorescent probe. 3-HIT treatment led to a robust increase in ROS accumulation, whereas CLU overexpression significantly decreased green fluorescence intensity, demonstrating an attenuation of oxidative stress (Figures 3I,J). Together, these results indicated that CLU exerted multifaceted cytoprotective effects—reducing inflammation, fibrosis, pyroptosis, and oxidative stress.

### 3.4 CLU binds to deubiquitination enzyme UCHL1 to promote NLRP3 degradation

**FIGURE 4:**
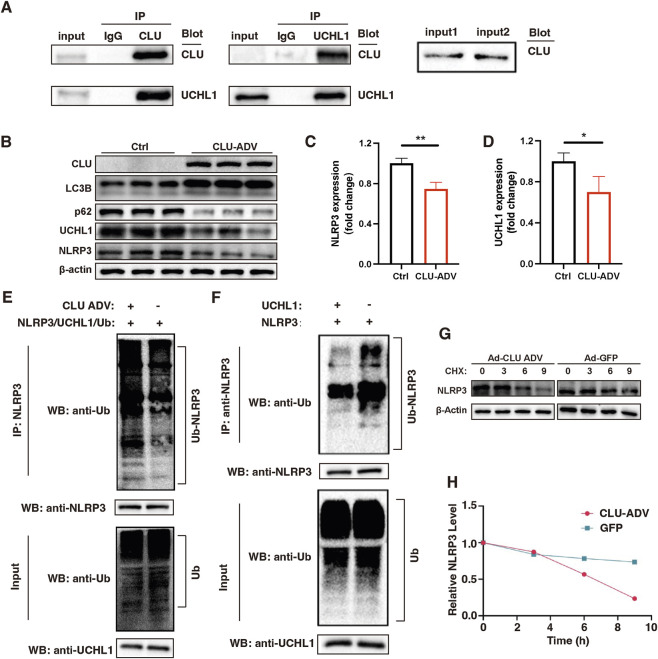
CLU interacted with UCHL1 and promoted NLRP3 degradation. **(A)** Western blot analysis of the interaction between CLU and UCHL1 detected by co-immunoprecipitation (Co-IP). **(B)** Western blot analysis of CLU, LC3B, p62, UCHL1, and NLRP3 in cells with or without CLU overexpression. β-actin was used as a loading control. Quantification of protein expression for **(C)** NLRP3 and **(D)** UCHL1. **(E)** The ubiquitination of NLRP3 promoted by CLU. **(F)** The ubiquitination of NLRP3 inhibited by UCHL1. **(G)** Western blot analysis of NLRP3 degradation after cycloheximide (CHX) treatment in CLU overexpressing cells. **(H)** Quantitative analysis of NLRP3 degradation.

To identify potential intracellular targets of CLU, an IP-MS assay was performed. Protein interactions identified by Co-IP coupled with LC-MS/MS were analyzed based on relative abundances. Fold changes (FC) were calculated by comparing the sample group with the IgG control group, and proteins with FC > 1.5 were considered as potential CLU-interacting candidates (Supplementary Table S1) Among these, UCHL1—a known deubiquitinating enzyme—was identified. Subsequent Co-IP confirmed a direct interaction between CLU and UCHL1 (Figure 4A). Notably, UCHL1 has been reported to modulate NLRP3 inflammasome activation (Ma, 2023). NLRP3, a critical component of the NOD-like receptor family, forms an inflammasome complex with ASC and pro-caspase-1 (Liu et al., 2023). Upon activation by danger signals, this complex facilitates caspase-1 activation and the subsequent maturation of pro-inflammatory cytokines IL-1β and IL-18, thereby promoting inflammation and pyroptotic cell death (Huang et al., 2021; Toldo and Abbate, 2024).

Given the known link between NLRP3 inflammasome activation and HFpEF (Seo et al., 2020), we investigated whether CLU modulates NLRP3 activity through UCHL1. Overexpression of CLU led to a reduction in both UCHL1 and NLRP3 protein levels, along with increased LC3B and decreased p62 expression, indicative of enhanced autophagic flux (Figures 4B–D). These results suggested that CLU may promote UCHL1 degradation through the autophagy-lysosome pathway. Furthermore, UCHL1 was found to reduce NLRP3 ubiquitination, while CLU disrupted this deubiquitination effect by binding to UCHL1, thereby enhancing NLRP3 ubiquitination (Figures 4E,F). CHX-chase assays further demonstrated that CLU overexpression accelerated the degradation of NLRP3, supporting its role in regulating NLRP3 protein stability (Figures 4G,H). Together, these results indicated that CLU suppressed NLRP3 inflammasome activation by promoting the autophagic degradation of UCHL1 and enhancing NLRP3 ubiquitination.

### 3.5 CLU peptide attenuated HFpEF progression in mice

**FIGURE 5:**
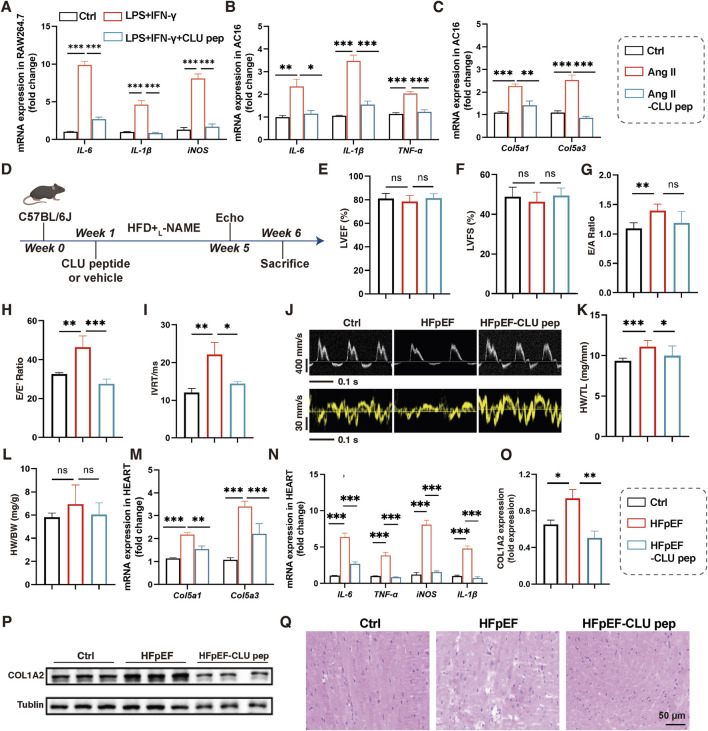
CLU peptide attenuated HFpEF progression in mice. **(A)** The mRNA expression of IL-6, IL-1β, and iNOS in Raw264.7 cells treated with LPS+IFN-γ and CLU peptide (n = 4). The mRNA expression of **(B)** IL-6, IL-1β, iNOS and **(C)** Col5a1, Col5a3 in AC16 cells treated with AngII and CLU peptide (n = 4). **(D)** Schematic of CLU peptide administration. Percentage of **(E)** LVEF, **(F)** LVFS, **(G)** E/A ratio **(H)** E/E′ ratio, and **(I)** IVRT of mice treatment with CLU peptide (n = 4). **(J)** Representative echocardiographic images of each group. **(K)** Ratio of heart weight to tibia length (HW/TL) and **(L)** heart weight to body weight (HW/BW) in mice (n = 4). mRNA expression of **(M)** Col5a1, Col5a3 and **(N)** IL-6, TNF-α, iNOS, IL-1β in mice hearts (n = 3). **(O,P)** COL1A2 protein expression in hearts detected by Western blot. **(Q)** Representative Hematoxylin-eosin staining images of hearts.

CLU peptide (the sequence: lvgrqleefl (D-amino acids)), is a peptide based on the structure of the CLU protein and has been reported to possess good anti-inflammatory activity (Moruz and Käll, 2017; Navab et al., 2005; Qi et al., 2018), but its effect on HFpEF has not been studied. First, the anti-inflammatory and antifibrotic capacity of CLU peptide on macrophages and cardiomyocytes was investigated. LPS+IFN-γ and Ang-II challenge led to significantly increased the mRNA expression of inflammatory genes (IL-6, IL-1β, iNOS, TNF-α) and fibrosis genes (col5a1, clo5a3) in Raw264.7 cells and AC16 cells, while CLU peptide treatment inhibited the mRNA expression of these genes (Figures 5A–C). Subsequently, the *in vivo* efficacy of CLU peptide against HFpEF was examined. Mice were exposed to HFD and _L_-NAME diet to induce HFpEF, and CLU peptide was administered at an oral dose of 5 mg/kg for 5 weeks (Figure 5D). Then, echocardiography was performed to determine the cardiac function of the mice. Heart rates of mice were monitored during echocardiographic measurements and consistently maintained within the physiological range of 400–500 bpm (Supplementary Figure S2C). Results showed that the LVEF and LVFS remained essentially unchanged (Figures 5E,F). The mice displayed a significant improvement in diastolic function after CLU peptide treatment, manifested by a decrease in the E/A, E/E′ and IVRT (Figures 5G–J). Meanwhile, the cardiac hypertrophy was also reduced (Figures 5K,L). The expression of myocardial fibrosis and inflammation related genes of heart were also detected. There were significant reductions in inflammation and fibrosis in the hearts of mice in the CLU peptide-treated group (Figures 5M,N). COL1A2 protein is an essential marker of cardiac fibrosis. It was found COL1A2 protein expression significantly increased in the hearts of mice challenged by HFD and _L_-NAME diet, indicating an accentuated cardiac fibrosis, while CLU peptide treatment reversed COL1A2 protein expression to normal levels (Figures 5O,P). Hematoxylin-eosin staining further confirmed that there was significant impairment of heart inflammation and fibrosis in the CLU peptide-treated group of mice (Figure 5Q). Therefore, these results demonstrated that CLU peptide attenuated the progression of HFpEF by reducing the level of inflammation and fibrosis.

## 4 Discussion

HFpEF is a complex and heterogeneous clinical syndrome characterized by systemic inflammation, myocardial fibrosis, and impaired diastolic function, for which effective therapeutic options remain limited (Redfield and Borlaug, 2023). Clusterin (CLU), a chaperone-like glycoprotein, has been shown in our previous studies to attenuate diabetes-associated atherosclerosis by inhibiting proinflammatory cytokine release and macrophage pyroptosis (Rodríguez-Rivera et al., 2021; Xuan et al., 2025). In the present study, we revealed a new regulatory mechanism by which CLU mitigates cardiac inflammation and fibrosis in HFpEF through modulation of the UCHL1–NLRP3 axis. These findings provided new insight into the biological function of CLU and highlight its therapeutic potential for this challenging condition.

Our key findings showed that CLU overexpression attenuated HFpEF-related phenotypes in mice, while hepatocyte-specific knockdown of CLU aggravated disease progression. Because circulating CLU is predominantly derived from the liver, we employed hepatocyte-specific CLU knockout mice to further verify its role in HFpEF progression. These results were consistent with prior observations that systemic inflammation and hepatic signaling pathways influence cardiac remodeling in HFpEF (Boulet et al., 2024; Schiattarella et al., 2019). Together, these results emphasized the importance of liver–heart crosstalk in HFpEF pathophysiology, suggesting that hepatic CLU served as a key regulator of systemic inflammatory homeostasis. Perturbations in hepatic signaling, such as impaired CLU production, can disrupt systemic inflammatory balance and thereby promote inflammasome activation in the heart. Importantly, our work identified a novel molecular mechanism whereby CLU interacted with the deubiquitinating enzyme UCHL1 and promoted the degradation of NLRP3, thereby suppressing inflammasome activation and the downstream inflammatory.

Previous studies have implicated NLRP3 inflammasome activation in the pathogenesis of HFpEF, linking metabolic stress and inflammation to myocardial stiffness and fibrosis (Cheng et al., 2023; Toldo and Abbate, 2024). Our study extended these findings by revealing that CLU regulated UCHL1-mediated NLRP3 deubiquitination, thereby modulating the post-translational control of inflammasome activation. This suggested that CLU may function not only as an extracellular chaperone but also as a modulator of intracellular proteostasis and inflammatory signaling, adding a novel regulatory layer to the current understanding of HFpEF pathogenesis.

Peptide mimetics of functional proteins have emerged as promising therapeutic agents for cardiovascular diseases, owing to their high target specificity, favorable safety profiles, and amenability to chemical modification (Mallart et al., 2021; Wolska et al., 2021). For instance, apelin-derived peptides have shown cardioprotective effects in heart failure models (Li J. et al., 2024). Building upon these advances, we evaluated the therapeutic potential of a clusterin-derived peptide mimetic, CLU-113, in the treatment of HFpEF. Although peptide-based therapies are often hindered by poor oral bioavailability and rapid enzymatic degradation (Verma et al., 2021; Wang et al., 2022), the incorporation of D-amino acids in CLU-113 significantly improved its proteolytic stability and oral bioavailability (Cooper et al., 2021; Navab et al., 2005). The observation that CLU-113 treatment alleviated cardiac inflammation and fibrosis in HFpEF mice supports the translational potential of targeting the CLU–UCHL1–NLRP3 pathway for therapeutic intervention.

However, our study has several limitations. Although we confirmed CLU’s interaction with UCHL1 and its downstream effect on NLRP3 degradation, the precise structural basis and dynamics of this interaction remain to be elucidated. While our animal data were promising, the translational relevance to human HFpEF patients’ needs further validation, including assessments in large-animal models and clinical samples. In addition, our study primarily focused on cardiac and hepatic CLU expression, but CLU is expressed in multiple organs, and systemic effects cannot be excluded. The HFpEF model was established using 5 weeks of HFD plus _L_-NAME, which was sufficient to reproduce early diastolic dysfunction, myocardial stiffness, and mild systemic inflammation. However, longer treatment may produce a more stable chronic phenotype, and future studies will extend the duration to further validate the model and therapeutic effects of CLU and its peptide derivative. Another limitation is that a standard positive control drug was not included in this study. Because our primary objective was to elucidate the mechanistic role of CLU and its peptide in HFpEF, we did not perform direct efficacy comparisons with established HFpEF therapies. Future work will incorporate clinically relevant positive control agents to better contextualize the therapeutic potential of CLU-based interventions. Furthermore, the future studies are needed to comprehensively characterize the oral pharmacokinetics of the CLU peptide and to quantify its tissue distribution, particularly its accumulation in the heart.

In conclusion, this study identified CLU as a novel regulator of NLRP3 inflammasome activity through UCHL1-mediated ubiquitination and establish its therapeutic potential in HFpEF. Future research should focus on further delineating the molecular interactions and exploring CLU-based therapies in clinical contexts.

## 5 Conclusion

In this study, we discovered that CLU overexpression can improve cardiac diastolic dysfunction, decreased inflammation, and fibrosis in HFpEF mice. CLU exhibited cardioprotective effects, suggesting its potential for the prevention and treatment of HFpEF. In contrast, knocking out CLU reduced the ejection fraction in HFpEF mice and exacerbated heart damage. We identified an interaction between CLU and the deubiquitinase UCHL1 by IP-MS. Notably, CLU overexpression suppressed UCHL1 expression, thereby enhancing NLRP3 ubiquitination and promoting its degradation, which led to reduced inflammasome activation and inflammation. Furthermore, a synthetic CLU-derived peptide exhibited potent preventive effects in HFpEF mice by alleviating inflammation and fibrosis. Collectively, our study revealed a critical role of CLU in HFpEF pathogenesis and offer novel insights into CLU-based therapeutic strategies.

## Data Availability

The datasets presented in this study can be found in online repositories. The names of the repository/repositories and accession number(s) can be found in the article/Supplementary Material.
