# Supplementary material for: Clusterin protects against HFpEF by inhibiting UCHL1-mediated NLRP3 deubiquitylation and inflammasome activation
Source: Front Pharmacol. 2026 Jan 13;16:1704023. doi: 10.3389/fphar.2025.1704023 (PMC12835627; doi:10.3389/fphar.2025.1704023)
Supplement: Supplementary file 1 [file Supplementaryfile1.docx]

**Clusterin protects against HFpEF by inhibiting UCHL1-mediated NLRP3 deubiquitylation and inflammasome activation**

Jiangling Yu^1†^, Xiaoxu Kang^2†^, Rui Chang^2^, Cheng Zhang^3^, Song Yang^2^, Lang Chen^1^, Xinbo Wang^4^, Bing Hu^1^, Zixuan Wang^3^, Lili Gong^1,4*^ and Lihong Liu^1,3,4*^

1 Institute of Clinical Medical Sciences, China-Japan Friendship Hospital, Capital Medical University, Beijing, China

2 Institute of Clinical Medical Sciences, China-Japan Friendship Hospital, Beijing, China

3 Department of Pharmacy, China-Japan Friendship Hospital, Beijing, China

4 China-Japan Friendship Hospital (Institute of Clinical Medical Sciences), Chinese Academy of Medical Sciences & Peking Union Medical College, Beijing, China

*Corresponding authors. Tel: +86 010-84205559 (Lihong Liu), +86 010-84205669 (Lili Gong).

E-mail addresses: llh-hong@outlook.com (Lihong Liu), gonglili@126.com (Lili Gong).

†The authors contributed equally to this work.

**Table S1 Primer sequences used for RT-qPCR.**

| **Gene** | **Primer** | **Sequence (5' -> 3')** |
| --- | --- | --- |
| Col5a1 | Forward Primer | TACAACGAGCAGGGTATCCAG |
|  | Reverse Primer | ACTTGCCATCTGACAGGTTGA |
| Col5a3 | Forward Primer | GTGGCCGTCAGCATAGATGG |
|  | Reverse Primer | TGAATGTCTCCCTCGAAAGTCTT |
| IL-6 | Forward Primer | ACTCACCTCTTCAGAACGAATTG |
|  | Reverse Primer | CCATCTTTGGAAGGTTCAGGTTG |
| IL-1β | Forward Primer | TTCGACACATGGGATAACGAGG |
|  | Reverse Primer | TTTTTGCTGTGAGTCCCGGAG |
| NLRP3 | Forward Primer | CGTGAGTCCCATTAAGATGGAGT |
|  | Reverse Primer | CCCGACAGTGGATATAGAACAGA |
| Caspase-1 | Forward Primer | TTTCCGCAAGGTTCGATTTTCA |
|  | Reverse Primer | GGCATCTGCGCTCTACCATC |
| GSDMD | Forward Primer | GAGTGTGGCCTAGAGCTGG |
|  | Reverse Primer | GGCTCAGTCCTGATAGCAGTG |
| TNF-α | Forward Primer | CCTGTAGCCCACGTCGTAG |
|  | Reverse Primer | GGGAGTAGACAAGGTACAACCC |

**Table S2 Fold changes (FC) of abundances between the sample and control (IgG) groups were calculated, and an FC > 1.5 was used as the threshold.**

|  | **Description** | **Fold Change** |
| --- | --- | --- |
| A0A087WW87 | Immunoglobulin kappa variable 2-40 OS=Homo sapiens OX=9606 GN=IGKV2-40 PE=3 SV=2 | 131.23 |
| P07305 | Histone H1.0 OS=Homo sapiens OX=9606 GN=H1-0 PE=1 SV=3 | 18.04 |
| P61626 | Lysozyme C OS=Homo sapiens OX=9606 GN=LYZ PE=1 SV=1 | 17.17 |
| P45880 | Voltage-dependent anion-selective channel protein 2 OS=Homo sapiens OX=9606 GN=VDAC2 PE=1 SV=2 | 11.18 |
| A0A0J9YX35 | Immunoglobulin heavy variable 3-64D OS=Homo sapiens OX=9606 GN=IGHV3-64D PE=3 SV=1 | 10.62 |
| P99999 | Cytochrome c OS=Homo sapiens OX=9606 GN=CYCS PE=1 SV=2 | 7.27 |
| Q96P48 | Arf-GAP with Rho-GAP domain, ANK repeat and PH domain-containing protein 1 OS=Homo sapiens OX=9606 GN=ARAP1 PE=1 SV=3 | 6.85 |
| O60869 | Endothelial differentiation-related factor 1 OS=Homo sapiens OX=9606 GN=EDF1 PE=1 SV=1 | 6.78 |
| P43490 | Nicotinamide phosphoribosyltransferase OS=Homo sapiens OX=9606 GN=NAMPT PE=1 SV=1 | 5.96 |
| Q86YZ3 | Hornerin OS=Homo sapiens OX=9606 GN=HRNR PE=1 SV=2 | 5.92 |
| Q9BZH6 | WD repeat-containing protein 11 OS=Homo sapiens OX=9606 GN=WDR11 PE=1 SV=1 | 5.86 |
| P04843 | Dolichyl-diphosphooligosaccharide--protein glycosyltransferase subunit 1 OS=Homo sapiens OX=9606 GN=RPN1 PE=1 SV=1 | 5.70 |
| P05155 | Plasma protease C1 inhibitor OS=Homo sapiens OX=9606 GN=SERPING1 PE=1 SV=2 | 5.59 |
| O95573 | Fatty acid CoA ligase Acsl3 OS=Homo sapiens OX=9606 GN=ACSL3 PE=1 SV=3 | 4.15 |
| Q7L7L0 | Histone H2A type 3 OS=Homo sapiens OX=9606 GN=H2AW PE=1 SV=3 | 4.11 |
| P23142 | Fibulin-1 OS=Homo sapiens OX=9606 GN=FBLN1 PE=1 SV=4 | 4.10 |
| P62942 | Peptidyl-prolyl cis-trans isomerase FKBP1A OS=Homo sapiens OX=9606 GN=FKBP1A PE=1 SV=2 | 4.00 |
| Q01081 | Splicing factor U2AF 35 kDa subunit OS=Homo sapiens OX=9606 GN=U2AF1 PE=1 SV=3 | 3.91 |
| P15924 | Desmoplakin OS=Homo sapiens OX=9606 GN=DSP PE=1 SV=3 | 3.70 |
| Q8IXR5 | Protein FAM178B OS=Homo sapiens OX=9606 GN=FAM178B PE=2 SV=3 | 3.45 |
| P05109 | Protein S100-A8 OS=Homo sapiens OX=9606 GN=S100A8 PE=1 SV=1 | 3.07 |
| B9A064 | Immunoglobulin lambda-like polypeptide 5 OS=Homo sapiens OX=9606 GN=IGLL5 PE=2 SV=2 | 3.03 |
| P68036 | Ubiquitin-conjugating enzyme E2 L3 OS=Homo sapiens OX=9606 GN=UBE2L3 PE=1 SV=1 | 2.89 |
| Q02413 | Desmoglein-1 OS=Homo sapiens OX=9606 GN=DSG1 PE=1 SV=2 | 2.80 |
| P05413 | Fatty acid-binding protein, heart OS=Homo sapiens OX=9606 GN=FABP3 PE=1 SV=4 | 2.73 |
| Q07955 | Serine/arginine-rich splicing factor 1 OS=Homo sapiens OX=9606 GN=SRSF1 PE=1 SV=2 | 2.67 |
| P09936 | Ubiquitin carboxyl-terminal hydrolase isozyme L1 OS=Homo sapiens OX=9606 GN=UCHL1 PE=1 SV=2 | 2.65 |
| P01023 | Alpha-2-macroglobulin OS=Homo sapiens OX=9606 GN=A2M PE=1 SV=3 | 2.46 |
| P62070 | Ras-related protein R-Ras2 OS=Homo sapiens OX=9606 GN=RRAS2 PE=1 SV=1 | 2.41 |
| P81605 | Dermcidin OS=Homo sapiens OX=9606 GN=DCD PE=1 SV=2 | 2.37 |
| P01857 | Immunoglobulin heavy constant gamma 1 OS=Homo sapiens OX=9606 GN=IGHG1 PE=1 SV=1 | 2.27 |
| P21291 | Cysteine and glycine-rich protein 1 OS=Homo sapiens OX=9606 GN=CSRP1 PE=1 SV=3 | 2.25 |
| P62805 | Histone H4 OS=Homo sapiens OX=9606 GN=H4C1 PE=1 SV=2 | 2.19 |
| P02771 | Alpha-fetoprotein OS=Homo sapiens OX=9606 GN=AFP PE=1 SV=1 | 2.14 |
| Q5QNW6 | Histone H2B type 2-F OS=Homo sapiens OX=9606 GN=H2BC18 PE=1 SV=3 | 2.13 |
| P01024 | Complement C3 OS=Homo sapiens OX=9606 GN=C3 PE=1 SV=2 | 2.06 |
| Q08554 | Desmocollin-1 OS=Homo sapiens OX=9606 GN=DSC1 PE=1 SV=2 | 2.03 |
| P61604 | 10 kDa heat shock protein, mitochondrial OS=Homo sapiens OX=9606 GN=HSPE1 PE=1 SV=2 | 2.03 |
| P55769 | NHP2-like protein 1 OS=Homo sapiens OX=9606 GN=SNU13 PE=1 SV=3 | 1.99 |
| P02768 | Albumin OS=Homo sapiens OX=9606 GN=ALB PE=1 SV=2 | 1.87 |
| Q14676 | Mediator of DNA damage checkpoint protein 1 OS=Homo sapiens OX=9606 GN=MDC1 PE=1 SV=3 | 1.86 |
| P04264 | Keratin, type II cytoskeletal 1 OS=Homo sapiens OX=9606 GN=KRT1 PE=1 SV=6 | 1.82 |
| P01834 | Immunoglobulin kappa constant OS=Homo sapiens OX=9606 GN=IGKC PE=1 SV=2 | 1.78 |
| Q9BUP0 | EF-hand domain-containing protein D1 OS=Homo sapiens OX=9606 GN=EFHD1 PE=1 SV=1 | 1.75 |
| P51911 | Calponin-1 OS=Homo sapiens OX=9606 GN=CNN1 PE=1 SV=2 | 1.73 |
| Q13835 | Plakophilin-1 OS=Homo sapiens OX=9606 GN=PKP1 PE=1 SV=2 | 1.73 |
| P16402 | Histone H1.3 OS=Homo sapiens OX=9606 GN=H1-3 PE=1 SV=2 | 1.72 |
| P40926 | Malate dehydrogenase, mitochondrial OS=Homo sapiens OX=9606 GN=MDH2 PE=1 SV=3 | 1.72 |
| Q8N884 | Cyclic GMP-AMP synthase OS=Homo sapiens OX=9606 GN=CGAS PE=1 SV=2 | 1.72 |
| O60701 | UDP-glucose 6-dehydrogenase OS=Homo sapiens OX=9606 GN=UGDH PE=1 SV=1 | 1.68 |
| P02751 | Fibronectin OS=Homo sapiens OX=9606 GN=FN1 PE=1 SV=5 | 1.68 |
| O00151 | PDZ and LIM domain protein 1 OS=Homo sapiens OX=9606 GN=PDLIM1 PE=1 SV=4 | 1.67 |
| P31949 | Protein S100-A11 OS=Homo sapiens OX=9606 GN=S100A11 PE=1 SV=2 | 1.66 |
| P40925 | Malate dehydrogenase, cytoplasmic OS=Homo sapiens OX=9606 GN=MDH1 PE=1 SV=4 | 1.64 |
| Q10567 | AP-1 complex subunit beta-1 OS=Homo sapiens OX=9606 GN=AP1B1 PE=1 SV=3 | 1.64 |
| P16401 | Histone H1.5 OS=Homo sapiens OX=9606 GN=H1-5 PE=1 SV=3 | 1.62 |
| Q16778 | Histone H2B type 2-E OS=Homo sapiens OX=9606 GN=H2BC21 PE=1 SV=3 | 1.62 |
| P47914 | 60S ribosomal protein L29 OS=Homo sapiens OX=9606 GN=RPL29 PE=1 SV=2 | 1.58 |
| Q15370 | Elongin-B OS=Homo sapiens OX=9606 GN=ELOB PE=1 SV=1 | 1.58 |
| P63241 | Eukaryotic translation initiation factor 5A-1 OS=Homo sapiens OX=9606 GN=EIF5A PE=1 SV=2 | 1.57 |
| Q5D862 | Filaggrin-2 OS=Homo sapiens OX=9606 GN=FLG2 PE=1 SV=1 | 1.57 |
| P11387 | DNA topoisomerase 1 OS=Homo sapiens OX=9606 GN=TOP1 PE=1 SV=2 | 1.54 |
| P13010 | X-ray repair cross-complementing protein 5 OS=Homo sapiens OX=9606 GN=XRCC5 PE=1 SV=3 | 1.53 |
| P0DUB6 | Alpha-amylase 1A OS=Homo sapiens OX=9606 GN=AMY1A PE=1 SV=1 | 1.52 |
| Q09666 | Neuroblast differentiation-associated protein AHNAK OS=Homo sapiens OX=9606 GN=AHNAK PE=1 SV=2 | 1.50 |


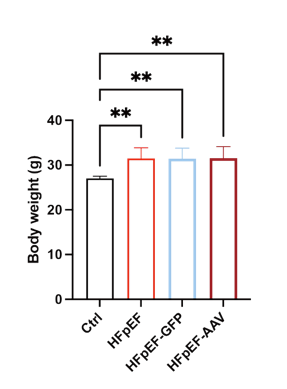


**Figure S1** Body weight of mice from different groups at week 5.


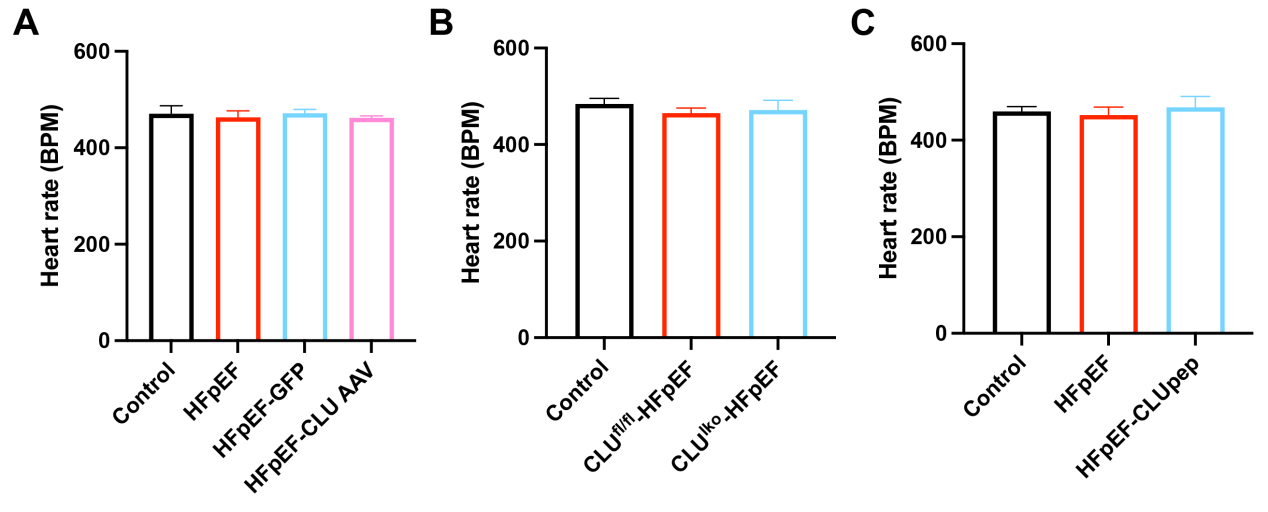


**Figure S2 Heart rate of mice measured during echocardiography.** Heart rate was recorded in each group at the time of echocardiographic assessment to ensure comparable physiological conditions across groups. (A) Mice used for evaluating the effects of CLU overexpression on HFpEF. (B) Mice used for evaluating the effects of liver-specific CLU knockout on HFpEF. (C) Mice used for evaluating the therapeutic effects of CLU peptide on HFpEF.

**
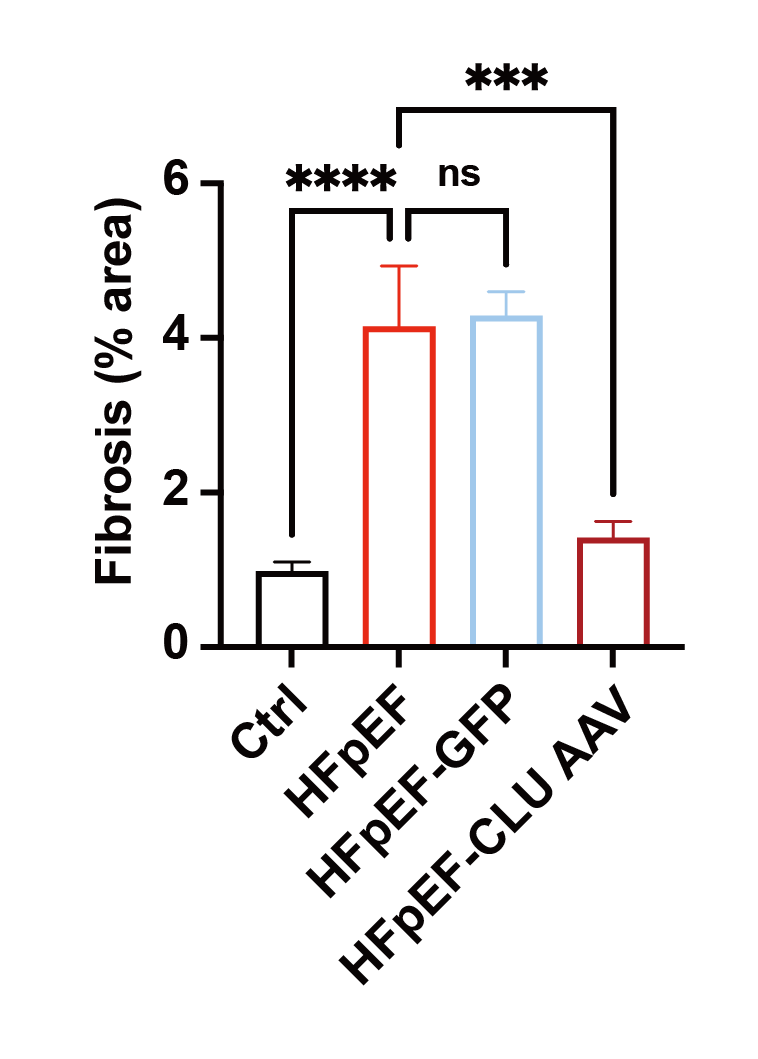
**

**Figure S3** Percentage of fibrosis area in Masson’s trichrome staining heart sections.

**
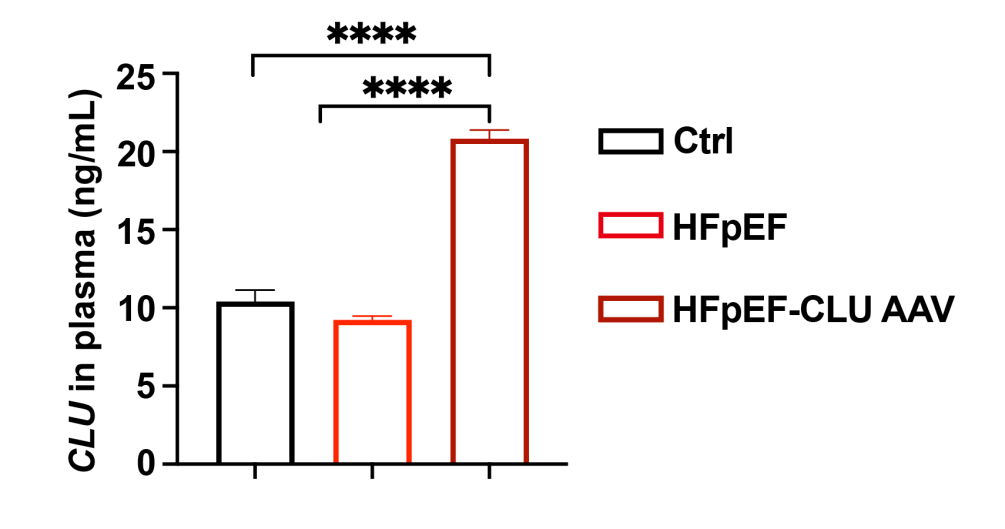
**

**Figure S4** CLU protein expression in plasma.

**
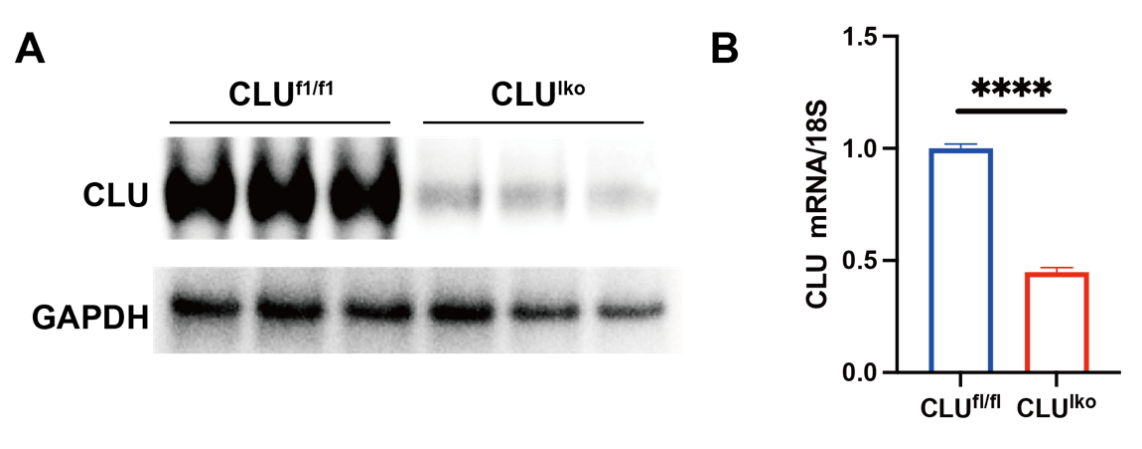
**

**Figure S5. (A)** Western blot images of CLU expression in the hearts of CLU^f1/f1^ and CLU^lko^ mice; (B) mRNA level of CLU in the hearts of CLU^f1/f1^ and CLU^lko^ mice.
